# Capturing alterations of intracellular–extracellular lactate distribution in the brain using diffusion-weighted MR spectroscopy in vivo

**DOI:** 10.1073/pnas.2403635121

**Published:** 2024-07-01

**Authors:** Sophie Malaquin, Rodrigo Lerchundi, Eloïse Mougel, Julien Valette

**Affiliations:** ^a^Laboratoire des Maladies Neurodégénératives, Université Paris-Saclay, Commissariat à l’Energie Atomique et aux Energies Alternatives (CEA), CNRS, Molecular Imaging Research Center (MIRCen), Fontenay-aux-Roses 92260, France

**Keywords:** NMR spectroscopy, lactate, brain metabolism, metabolite diffusion, compartmentation

## Abstract

While the intracellular–extracellular distribution of lactate has been suggested to play a critical role in the healthy and diseased brain, tools are lacking to noninvasively probe lactate in intracellular and extracellular spaces. Here, we show that, by measuring the diffusion of lactate with diffusion-weighted magnetic resonance (MR) spectroscopy in vivo and comparing it to the diffusion of purely intracellular metabolites, noninvasive quantification of extracellular and intracellular lactate fractions becomes possible. More specifically, we detect alterations of lactate diffusion in the APP/PS1 mouse model of Alzheimer’s disease. Data modeling allows quantifying decreased extracellular lactate fraction in APP/PS1 mice as compared to controls, which is quantitatively confirmed with implanted enzyme-microelectrodes. The capability of diffusion-weighted MR spectroscopy to quantify extracellular–intracellular lactate fractions opens a window into brain metabolism, including in Alzheimer’s disease.

Once considered as waste, lactate has emerged as a central metabolic substrate and signaling molecule (1). It has been proposed to be tightly associated with glutamatergic neurotransmission via the astrocyte-to-neuron lactate shuttle (ANLS), where lactate flows from astrocytes to the extracellular space and then to neurons (2). Specific lactate compartmentation is a prerequisite for shuttling, as a concentration gradient is required for lactate to effectively flow through monocarboxylate transporters (3). More recently, lactate compartmentation and shuttling have been proposed to be of critical importance in synaptic plasticity and long-term memory (4). Furthermore, dysregulations of lactate metabolism have been suggested to play a crucial role in Alzheimer’s disease, which is now considered by some as a metabolic disease (5, 6). However, these notions remain challenged and difficult to investigate, which is largely due to the lack of noninvasive tools to quantify lactate in the different compartments, starting with intracellular versus extracellular spaces.

Here, we show, in the amyloid precursor protein / presenilin 1 (APP/PS1) mouse model of Alzheimer’s disease, that diffusion-weighted magnetic resonance spectroscopy (dMRS) in vivo allows capturing variations of the intracellular–extracellular distribution of brain lactate, by exploiting the different diffusion properties in both compartments. Data modeling, based on solid assumptions derived from recent experimental findings, allows quantifying variations of intracellular–extracellular lactate fractions, which are in line with “ground truth” variations of extracellular lactate as measured with implanted enzyme-electrodes.

## Results and Discussion

In dMRS, the signal is made sensitive to molecular displacement during a given “diffusion time,” by application of some “diffusion-weighting” (denoted *b*): To put it simply, the larger *b* and the average displacement during the diffusion time, the weaker the dMRS signal is. Endogenous metabolites can be considered as molecular probes randomly exploring cells from within, over micrometric scales, under the effect of thermal motion. Unlike lactate, most MRS-visible metabolites are almost exclusively intracellular, and their specific dMRS signal attenuation (with significant signal remaining at high *b*) reflects restricted motion within cells, which may be used to assess cell microstructure (7). In contrast, extracellular diffusion was recently demonstrated to be fast (and approximately monoexponential), leading to quick signal disappearance as *b* is increased (8). Hence, intracellular and extracellular lactate pools may in theory be separated when measuring signal attenuation from low to high *b*.

**Fig. 1.**
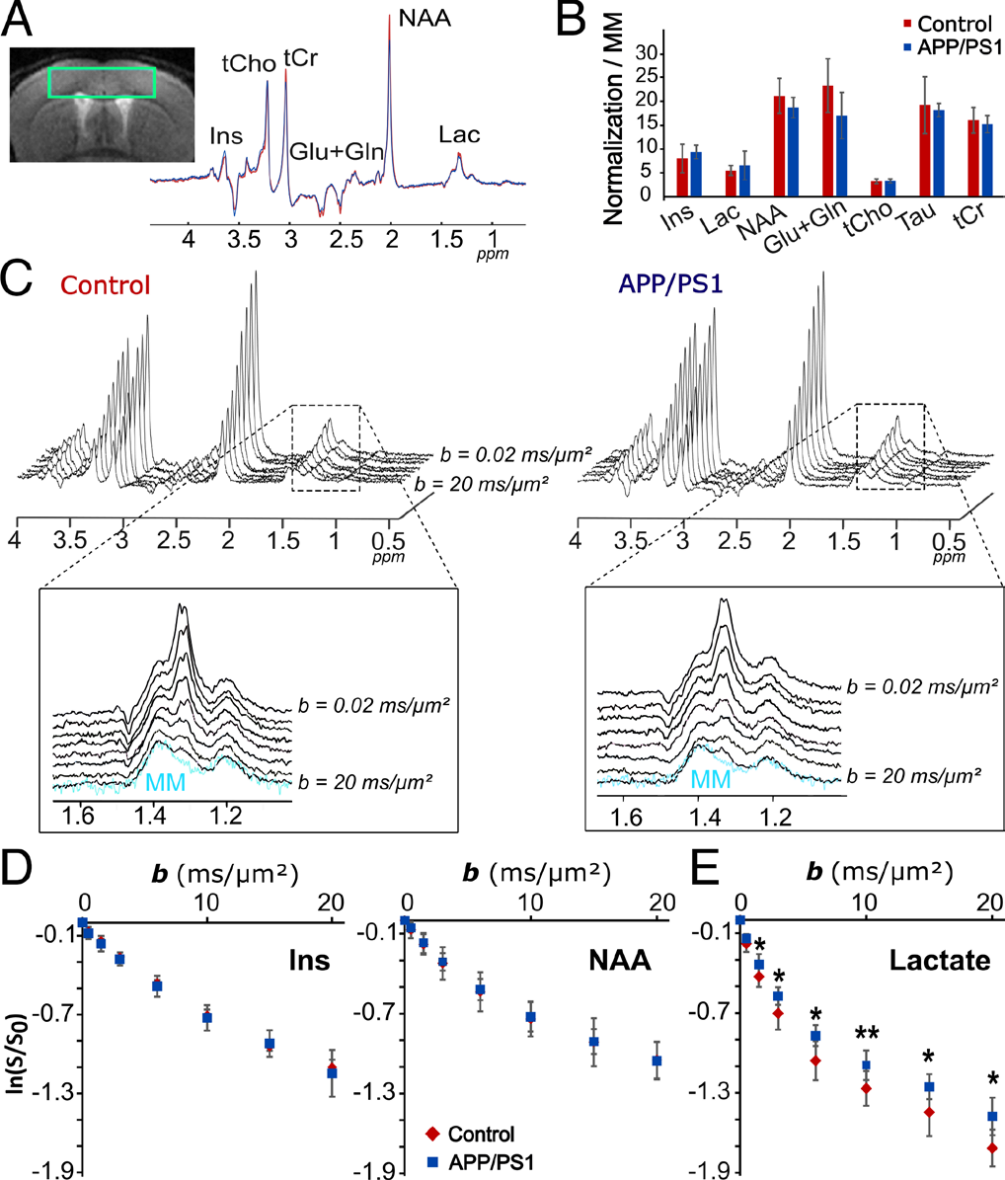
Diffusion-weighted MR spectroscopy acquisitions in the mouse brain. (*A*) Position of the 15-µL voxel in the mouse cortex, and spectra acquired at the lowest *b*-value (averaged here for display over the five mice of each group. (*B*) No significant variation of metabolite concentration (normalized relative to macromolecules) was detected between APP/PS1 and control mice. (*C*) Stackplots of diffusion-weighted spectra averaged over the five mice of both groups, and zoom around the lactate peak, with contribution of macromolecules in blue (the macromolecule spectrum shown here being the spectrum averaged across all animals in both groups). (*D*) Diffusion-weighted signal attenuation of intracellular metabolites Ins and NAA in both groups, showing no significant difference. (*E*) In contrast, lactate signal is less attenuated in APP/PS1 mice. Error bars stand for SD as calculated across the five animals in each group. Statistical difference between groups was assessed with unpaired Student’s *t* tests (*P < 0.05, **P < 0.01).

We performed dMRS in a cortical voxel (Fig. 1*A*) of five ~15-mo-old APP/PS1 mice and five age-matched controls, for *b* ranging from 0.02 to 20 ms/µm^2^ (at constant diffusion time *t_d_* = 53.2 ms), using a sequence specifically designed for optimal measurement of lactate diffusion (9), with an interleaved acquisition scheme making the measurement insensitive to slow concentration variations potentially occurring during the experiment. Anesthesia was maintained with 1% isoflurane in a 1:1 air:O_2_ mixture. Spectra acquired at the lowest *b* were used to assess potential variations of metabolite concentrations, including lactate, which were found to be nonsignificant (Fig. 1*B*). Overall, signal attenuations for intracellular metabolites (N-acetylaspartate NAA; total creatine, tCr; choline compounds, tCho; *myo*-inositol, Ins) were identical between both groups (Fig. 1 *C* and *D*), meaning that cellular microstructure as probed by dMRS was not altered in APP/PS1. In contrast, for lactate, dMRS revealed less pronounced signal attenuation in APP/PS1 (Fig. 1*E*).

**Fig. 2.**
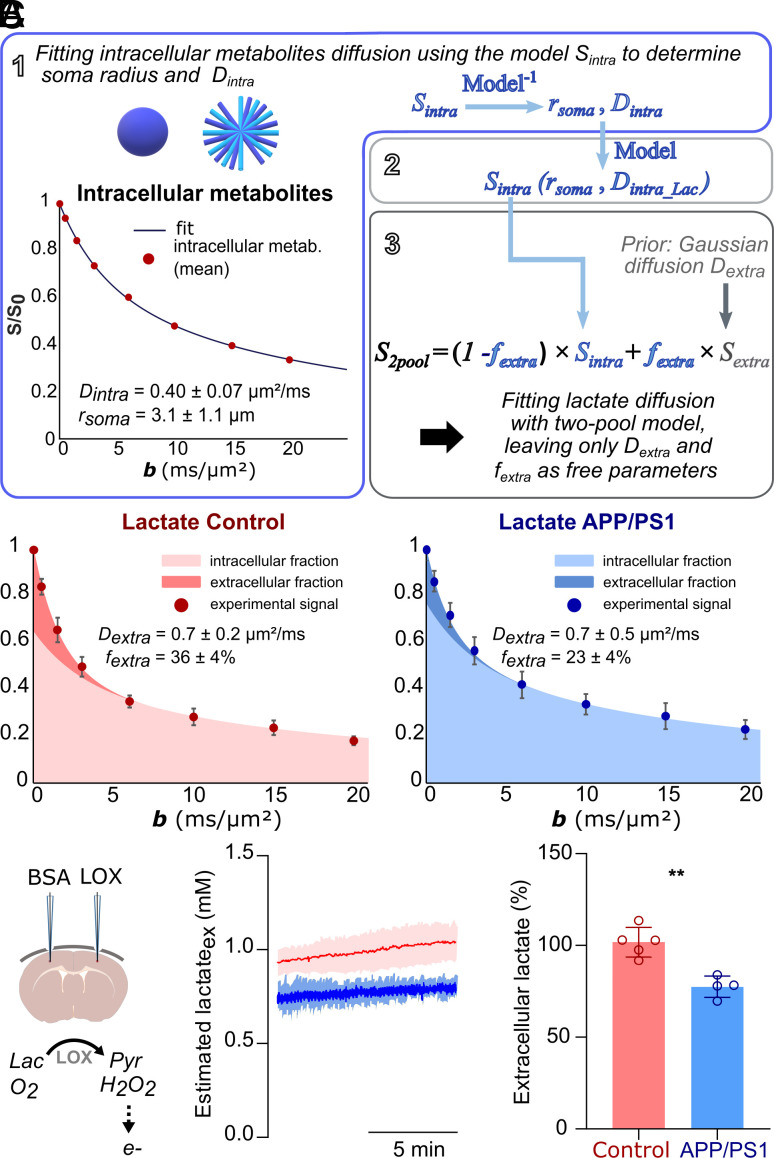
Quantifying extracellular–intracellular lactate fractions. (*A*) Schematics of the modeling pipeline. In the first step, the signal of intracellular metabolites NAA and Ins is averaged and fitted using a model of sticks and spheres to extract the intracellular diffusivity *D_intra_* and soma radius *r_soma_* (*D_intra_* = 0.40 ± 0.07 µm^2^/ms and *r_soma_* = 3.1 ± 1.1 µm for current experimental data). In the second step, the intracellular signal attenuation of lactate is computed in the same geometries, after correcting *D_intra_* for the larger free diffusivity of lactate. In the third step, experimental lactate signal attenuation is fitted using a two-pool model, taking into account prior knowledge about intracellular lactate signal attenuation as computed in previous steps and assuming Gaussian diffusion in the extracellular space. (*B*) Fit of lactate signal attenuation in both groups, showing relative contributions of intracellular and extracellular pools to the signal. While extracellular diffusion *D_extra_* does not vary, extracellular lactate fraction *f_extra_* exhibits a ~one-third decrease in the APP/PS1 group. (*C*) Electrochemical detection of extracellular lactate using implanted enzyme-electrodes confirms decreased extracellular lactate concentration in APP/PS1 (**P < 0.01, unpaired Student’s *t* tests). Note that, while “absolute” concentrations are shown for the in vivo curves following in vitro calibrations, only relative variation is reported, because it is the most reliable.

Because no alteration of intracellular diffusion occurs in APP/PS1, slower lactate diffusion has to find its origin in the extracellular space and may be due either to slower extracellular diffusion or to smaller extracellular fraction of lactate. We decided to perform diffusion modeling to investigate this point, which required developing some original modeling strategy. In one pioneer work published more than two decades ago (10), it was proposed to estimate intracellular–extracellular lactate fraction using phenomenological biexponential modeling. To do that, the authors had to assume that, while extracellular lactate diffusion was monoexponential, intracellular lactate would exhibit the same biexponential behavior as MRS-visible intracellular metabolites (i.e., identical slow/fast fractions). This is unlikely because, due to its smaller size, lactate diffusivity is ~30% larger than MRS-visible intracellular metabolites. To explicitly account for the larger diffusivity of lactate when predicting its diffusion signal in the intracellular space, we decided to perform biophysical modeling. In the end, modeling broadly consisted in three steps (Fig. 2*A*):

i) The determination of cellular microstructure, by fitting measured intracellular metabolite diffusion (NAA and Ins pooled together, see *SI Appendix*) with a model of diffusion in sticks and spheres (whose diameter is let as free parameter), e.g., as in the SANDI model (11), which we have shown to adequately account for intracellular metabolite diffusion up to large *b* values (12).
ii) The prediction of intracellular lactate diffusion attenuation *S_intra_Lac_*(*b*) in these spheres and sticks, taking into account its larger diffusivity as compared to intracellular metabolites.
iii) The determination of lactate extracellular diffusion coefficient (*D_extra_*) and extracellular fraction *f_extra_*, by fitting experimental lactate attenuation with *f_extra_*×exp(-*b*×*D_extra_*) + (1-*f_extra_*) × *S_intra_Lac_*(*b*).

Note that lactate signal is represented by the sum of intracellular and extracellular pools treated independently, which relies on the assumption of negligible exchange during the diffusion time. This is substantiated by recent measurements showing that—like nonexchanging intracellular metabolites, but unlike water—lactate Kurtosis keeps increasing with the diffusion time (13): This is a sign that pools do not equilibrate through cell membranes, which still act as restriction barriers. Further details and justifications about the model are given in *SI Appendix*.

In the end, data modeling yielded nonsignificant variation of *D_extra_*, but significant ~one-third decrease of *f_extra_* (23 ± 4% in APP/PS1 versus 36 ± 4% in control) (Fig. 2*B*). In the context of stable total lactate concentration in APP/PS1, this means that an equivalent decrease in extracellular lactate concentration is expected. Importantly, these results appeared to be quite robust to changes in the model of microstructure (i.e., changing the volume ratio between sticks and spheres, or using non-zero-diameter cylinders only), as long as the model parameters, namely diameter and NAA and Ins intracellular diffusivity, adequately described the diffusion of intracellular metabolites. However, accounting for the larger diffusivity of intracellular lactate appeared to be more important, reaffirming the necessity of biophysical modeling to properly predict intracellular lactate signal attenuation based on the diffusion of intracellular metabolites (*SI Appendix*).

To confirm variation of extracellular lactate content, we performed electrochemical quantification of extracellular lactate using implanted enzyme-electrodes (14). Experiments were carried out on four APP/PS1 and five controls (ranging from 12 to 15 mo). The determination of lactate absolute concentration by such microelectrodes is influenced by the microenvironment directly surrounding the electrode tip. Tissue damage during implantation, along with variations in oxygen levels—particularly under anesthesia conditions—poses challenges in establishing absolute values through in vitro calibrations. This may result in the underestimation of the actual lactate concentrations as compared to phantoms (15); hence, relative variations (in percent) are the most reliable. Under anesthesia conditions similar to dMRS experiments, extracellular lactate concentration was measured to decrease by 24 ± 2% in APP/PS1 (Fig. 2*C*), which is in good agreement with the ~one-third decrease of *f_extra_* (and thus equivalent decrease of extracellular lactate concentration in a context of stable total lactate concentration) as estimated from dMRS.

One possible confound might be some potential different sensitivity to anesthesia or to O_2_ levels in APP/PS1 mice, which may specifically alter intracellular–extracellular lactate distribution. To assess the possibility of such bias, we performed a set of complementary experiments to measure lactate diffusion in control mice under two different conditions (1% isoflurane in a 1:1 air:O_2_ mixture versus 1.3% isoflurane in a 1:4 air:O_2_ mixture). No significant difference was measured, which is strong evidence that different sensitivity to anesthesia/O_2_ cannot cause detectable remodeling of lactate distribution. Generally speaking, potential variations of inhaled O_2_ are unlikely to be a confound, since the high O_2_ levels used in our experiments ensure that O_2_ is not rate-limiting for oxidative metabolism. Hence, decreased extracellular lactate concentration certainly points toward metabolic remodeling in APP/PS1 mice. Rather than increased neuronal uptake, it may indicate reduced ANLS, with less lactate released from astrocytes and transiting through the extracellular space, resulting in a weaker concentration gradient from extracellular space to neurons, in line with earlier reports of extracellular lactate concentration correlating with energy metabolism and brain activity (4, 16, 17).

In conclusion, we have shown that dMRS is able to noninvasively capture alterations of lactate intracellular–extracellular distribution in the brain. Data modeling even allows quantifying variation of intracellular–extracellular lactate fractions, which is in line with “ground truth” as measured with implanted enzyme-electrodes. While this work was performed in the rodent brain, the same dMRS approach may potentially be used in Humans. This opens a new window into brain metabolism and ANLS in the healthy and diseased brain, including in Alzheimer’s disease.

## Materials and Methods

See *SI Appendix*.

## Supplementary Material

Appendix 01 (PDF)

## Data Availability

NMR spectra, quantified metabolite signals and enzyme-electrode recording data have been deposited in Zenodo (18).
