# Supplementary material for: Capturing alterations of intracellular–extracellular lactate distribution in the brain using diffusion-weighted MR spectroscopy in vivo
Source: Proc Natl Acad Sci U S A. 2024 Jul 1;121(28):e2403635121. doi: 10.1073/pnas.2403635121 (PMC11252949; doi:10.1073/pnas.2403635121)
Supplement: Supplementary file 1 — Appendix 01 (PDF) [file pnas.2403635121.sapp.pdf]

PNAS

Supporting Information for

Capturing alterations of intracellular-extracellular lactate distribution in the brain using diffusion-weighted MR spectroscopy *in vivo*

Sophie Malaquin, Rodrigo Lerchundi, Eloïse Mougel, Julien Valette

Corresponding author: Julien Valette
Email: julien.valette@cea.fr

This PDF file includes:

Supporting text
SI References

Supporting Text

Animal experimentation

Animal procedures were performed in accordance with the French regulation (EU directive 86/609 – French Act Rural Code R214–87 to 131). The animal facility was approved by veterinarian inspections (authorization n°B92–032-02) and complied with Standards for Humane Care and Use of Laboratory Animals of the Office of Laboratory Animal Welfare (OLAW – n°#A5826–01). All procedures received approval from the local ethical committee (Comité d'Ethique en Expérimentation Animale CEA) and the French Ministry of Research (APAFIS #32951-2021091411272817 v1).

Magnetic resonance experiments

Mice were scanned on an 11.7 T scanner (Bruker, Ettlingen, Germany) with maximal gradient strength G_{max}=752 mT/m on each axis, using a quadrature surface cryoprobe for transmission and reception. Anesthesia was induced with 4% isoflurane and maintained at 1% in a 1:1 air:oxygen mixture. Respiratory rate was continuously monitored using PC SAM software (Small Animal Instruments, Inc., Stony Brook, NY). Mouse body temperature was monitored with an endorectal probe and maintained at 37°C with regulated water flow. Signal was acquired in a 5×1×3-mm^3 spectroscopic volume of interest positioned in the cortex, using a home-made SE-LASER sequence and a spectrally-selective refocusing pulse (50-ms duration, 1800-Hz bandwidth, ~100-Hz transition band) to cancel the effect of J-modulation on the 1.3-ppm lactate resonance, elaborating on the approach introduced in (1). Residual water signal was suppressed using a VAPOR (VAriable Power radiofrequency pulses with Optimized Relaxation delays) module (2). For each mouse the following measurements were performed: first, a macromolecule (MM) spectrum was acquired during 17 minutes using a double inversion recovery module (3, 4) (TI_1/TI_2/TR=770/2200/4000 ms, 256 averages) positioned prior to the SE-LASER sequence and applying *b*=10 ms/µm² to reduce residual metabolite peaks. Spectra at different *b* values (*b*=0.02, 0.5, 1.5, 3.02, 6, 10, 15, and 20 ms/µm²) were then acquired (diffusion gradient duration δ=3.1 ms, diffusion time t_d=53.2 ms, TE/TR=83.4/2000 ms, 256 repeats). Importantly, *b*-values were acquired in an interlaced manner, i.e. *b* was changed for each repetition so that the whole *b*-value range was covered every 16 seconds, to avoid bias due to potential variations of metabolite concentration occurring over longer time-scales. Furthermore, to avoid bias due to potential diffusion anisotropy in the voxel, powder averaging was performed, i.e. diffusion gradient orientation was modified for each cycle of *b*-values to uniformly cover the surface of a sphere. Specifically, 16 gradient directions were generated using the Bruker module, as used in their commercial DTI sequence, that we plugged inside our dMRS sequence. This powder-averaging cycle was itself repeated 16 times, corresponding to 256 individual scans for each *b*-value. In the end, total duration for metabolite diffusion measurement was ~1h15min.

Scan-to-scan phase and frequency correction of individual repetitions were performed using Matlab: frequency was first corrected by non-linear least squares (*lsqnonlin* function) on the magnitude signal in the spectral domain, over a region spanning metabolite peaks. Phase was then corrected using the same function on the real part in the spectral domain. Frequency was corrected one last time, again on the absolute signal. Corrected spectra were then averaged to get one spectrum per *b*-value for each animal, before LCModel analysis incorporating macromolecule experimental spectrum in the basis-set (5).

Note that, because MM spectra were very stable between both groups, in terms of profile and amplitude, the MM spectrum included in LCModel basis-set was the average of MM spectra acquired across all animals of both groups. Due to this stability between groups, we also decided to report metabolite concentrations (as quantified at *b*=0.02 ms/µm²) normalized relative to MM content, which we considered to be a relevant and stable marker of cellularity (similar in spirit to normalization relative to dried tissue weight, as usually done in metabolomics analysis), less prone

to bias than normalization relative to total creatine or to water. In the end, estimated concentrations as normalized to MM are 5.3±1.2 in controls *versus* 6.4±3.0 in APP/PS1 (mean±s.d.), revealing no significant difference.

Because normalization relative to total creatine or water remains more conventional, we also provide lactate concentrations as estimated:

- Relative to tCr (in arbitrary unit): 0.34±0.07 in controls *versus* 0.43±0.22 in APP/PS1 (no significant difference).
- Relative to water signal as measured by peak integration on non-water-suppressed spectra (in arbitrary unit): 19.3±2.5 in controls *versus* 23.8±11.0 in APP/PS1 (no significant difference).

Neither T_1 nor T_2 correction was performed in the above estimates.

Possible confounds due to anesthesia / O_2

Additional experiments were performed in five control mice under two different anesthesia/O_2 conditions to assess whether anesthesia or oxygen levels may induce some variations of lactate distribution and hence bias comparison between groups (should groups exhibit different sensitivities to anesthesia or O_2).

After initial induction as described in the previous section, the following protocol was applied:

- 45 minutes of dMRS under 1.3% isoflurane in a 1:4 air:O_2 mixture.
- 10-minute transition to the same conditions as for the APP/PS1 *versus* control experiments (1% isoflurane in a 1:1 air:O_2 mixture).
- 45 minutes of dMRS under those conditions.

The same dMRS acquisition scheme was used as for the APP/PS1 *versus* control experiments, except that only four *b*-values (*b*=0.02, 3.02, 10 and 20 ms/µm²) were acquired to keep the duration of the experiment acceptable.

Although both conditions do not represent extreme variations of isoflurane and O_2 content, they are presumed large enough to represent any potential different sensitivities to isoflurane and O_2 between APP/PS1 and control mice.

In the end, Student's t-tests (even paired t-tests) reveal no significance difference in signal attenuation at any *b*-value between both conditions. It is worth noting that lactate concentration, as estimated from spectra at the lowest *b*, did not exhibit significant variation (either normalized relative to MM, or in absolute values).

Diffusion modeling

Signal attenuation was averaged for NAA and Ins across both groups, because these two intracellular metabolites were reliably quantified (LCModel's Cramér-Rao Lower Bounds <5% at all *b*-values) and, unlike tCr or tCho, they are not "mixed" pools, so that their free diffusion coefficient can be reliably determined in phantoms (see below). Average intracellular signal attenuation was then fitted with Matlab, using the following equation as described in (6) (setting a null-diameter for fibers, i.e. making them "sticks", and letting r_{soma} and D_{intra} as free parameters):

$$S_{intra}(b) = 0.8\times S_{fiber}(D_{intra},b) + 0.2\times S_{soma}(r_{soma},D_{intra},b) \qquad [1]$$

In the above expression, the 80%:20% fiber:soma volume fractions were taken from (6) as determined on actual cells.

Lactate signal was then fitted using (considering that diffusion in the extracellular space is approximately mono-exponential):

$S_{Lac}(b) = f_{extra}\times\exp(-b\times D_{extra})+(1-f_{extra})\times S_{intra\_Lac}(b)$ [2]

Where the expression for S_{intra_Lac} is similar to Eq.[1]:

$S_{intra\_Lac}(b) = 0.8\times S_{fiber}(D_{intra\_Lac},b) + 0.2\times S_{soma}(r_{soma},D_{intra\_Lac},b)$ [3]

In Eq.[2], f_{extra} and D_{extra} are left as free parameters, while D_{intra_Lac} in Eq.[3] is instead determined based on the intracellular diffusivity D_{intra} of intracellular metabolites as estimated from Eq.[1], and taking into account the intrinsically larger free diffusivity of lactate: $D_{intra\_Lac}=D_{intra}\times D_{free}(\text{Lac})/D_{free}(\text{intra})$, where D_{free} is the free diffusion coefficient as measured in phantoms (i.e. D_{free}(intra) is the average of D_{free}(Ins)=0.76 µm²/ms and D_{free}(NAA)=0.75 µm²/ms; and D_{free}(Lac)=0.99 µm²/ms).

All uncertainties were estimated using Monte Carlo simulations (n=1000 runs), and statistical significance was evaluated using permutation tests, as described in (7).

In Eq.[2], extracellular and intracellular lactate pools are treated independently, which relies on the assumption of slow exchange, i.e. the proportion of lactate exchanged during the diffusion time is small. We are aware of only a few works attempting to quantify lactate exchange, most of them using hyperpolarized ^{13}C, and all of them in cancer cells (8-11). Taking the “worst case scenario” in those works, corresponding to the fastest exchange rate (~0.3 s^{-1} (9), i.e. 30% of the pool being transported every second), the lactate fraction being transported during the diffusion time (~50 ms) is less than 2%, which can be considered as negligible.

While the above exchange rates were estimated in cancer cell cultures, we are not aware of such estimates in the living brain. However, previous dMRS works can be very insightful. We indeed showed in the mouse brain that Kurtosis (which describes how diffusion-weighted signal attenuation deviates from mono-exponentiality) time-dependence is very different for intracellular metabolites and water (12): while Kurtosis of intracellular metabolites keeps increasing up to the maximal diffusion time reached in that work (500 ms), water Kurtosis rapidly decreases, which is a sign of fast exchange (i.e. cell membranes can less and less be considered as barriers to water diffusion). It turns out that lactate Kurtosis exhibits similar behavior as intracellular metabolites, i.e. it keeps increasing (though slightly) with the diffusion time (13). Hence, one concludes that the largest fraction of the lactate pool has not yet been transported in 500 ms. This ensures that, in the context of the present study where the diffusion time is ~50 ms only, the exchange is negligible.

Sensitivity of estimated extracellular lactate fractions to intracellular diffusion model

To assess to what extent estimated extracellular lactate fraction depends on the biophysical model used to describe intracellular diffusion, the whole modeling pipeline as described above was iterated using different expressions for S_{intra}, corresponding to the models described below. All uncertainties were estimated using Monte Carlo simulations (n=500 runs).

1) 100% soma

For intracellular metabolite modeling we obtain r_{soma}=9.4±0.1 µm and D_{intra}=0.086±0.003 µm²/ms.
Then, fitting lactate signal, we obtain D_{extra}=1.0±0.3 µm²/ms in controls *versus* 0.9±0.5 µm²/ms in APP/PS1, and f_{extra}=36±4% in controls *versus* 24±4% in APP/PS1, i.e. very similar f_{extra} as for 80%:20% sticks:soma volume fractions.

2) 50%:50% sticks:soma volume fractions

For intracellular metabolite modeling we obtain r_{soma}=17.3 µm±0.5 µm and D_{intra}=0.144 ± 0.006 µm²/ms.
For lactate we then get D_{extra}=1.0±0.4 µm²/ms in controls *versus* 1.0±1.9 µm²/ms in APP/PS1, and f_{extra}=33±4% in controls *versus* 21±4% in APP/PS1, i.e. again very similar f_{extra} as for 80%:20% sticks:soma volume fractions.

3) 100% sticks

For intracellular metabolite modeling we obtain D_{intra}=0.33±0.02 µm²/ms. For lactate we then get D_{extra}=0.8±0.3 µm²/ms in controls *versus* 0.8±1.6 µm²/ms in APP/PS1, and f_{extra}=34±4% in controls *versus* 21±4% in APP/PS1, i.e. again very similar f_{extra} as for 80%:20% sticks:soma volume fractions.

4) <u>100% non-zero-diameter cylinders</u>

In this additional microstructural scenario, sticks were replaced by cylinders of non-zero diameter, the latter being left as free parameter, e.g. as described in one of our early modeling work (14).

For intracellular metabolite modeling we obtain $r_{cylinder}$=0.03±0.47 µm and D_{intra}=0.32±0.03 µm²/ms (i.e. very close to the 100%-stick model). For lactate we then get D_{extra}=0.8±0.3 µm²/ms in controls *versus* 0.8±1.5 µm²/ms in APP/PS1, and f_{extra}=34±4% in controls *versus* 20±4% in APP/PS1, again very similar f_{extra} as for 80%:20% sticks:soma volume fractions.

5) <u>Effect of changing D_{intra_Lac}</u>

Finally, we wanted to assess the effect of intracellular lactate diffusivity in the “canonical” 80%:20% sticks:soma microstructural model. Instead of rescaling D_{intra_Lac} to account for the intrinsically larger free diffusivity of lactate, i.e. $D_{intra\_Lac}=D_{intra}\times D_{free}$(Lac)/$D_{free}$(intra), we imposed intracellular lactate to have the same diffusivity as estimated for intracellular metabolites: $D_{intra\_Lac}=D_{intra}$=0.40 µm²/ms. When doing so, we get D_{extra}=0.7±0.2 µm²/ms in controls *versus* 0.6±0.2 µm²/ms in APP/PS1, and f_{extra}=42±4% in controls *versus* 30±4% in APP/PS1, i.e. quite larger than f_{extra} obtained with the 80%:20% sticks:soma model.

This effect is still exacerbated when imposing D_{intra_Lac} to be 50% lower than the values used in the manuscript, i.e. imposing D_{intra_Lac}=0.27 µm²/ms, which yields D_{extra}=0.6±0.1 µm²/ms in controls *versus* 0.5±0.1 µm²/ms in APP/PS1, and f_{extra}=50±4% in controls *versus* 40±3% in APP/PS1.

Recapitulating all these simulations, we conclude that the estimation of lactate distribution appears to be very robust to the microstructural model, but sensitive to the intracellular diffusivity. Our explanation is the following: as long as the model, even somehow inaccurate, has parameter values adequately describing signal attenuation for purely intracellular metabolites, the model can adequately enough predict intracellular lactate signal attenuation, provided D_{intra_Lac} is well parameterized.

Enzyme-electrodes

Lactate oxidase-based microelectrode biosensors were provided by the “BioELectrochimie In Vivo” platform from Lyon Neuroscience Research Center (Lyon, France). *In vitro* calibration of the biosensors included exposing them to increasing concentrations of lactate in PBS at 34-37°C, both before and after each experiment. Functionality and insensitivity was confirmed by exposure to H_2O_2 (5 µM), lactate (100 µM) and the electroactive molecule serotonin (20 µM) respectively.

Thirty minutes before the procedure, a subcutaneous injection of buprenorphine (0.075 mg/kg) was administered to provide optimal pain management. Anesthesia was then induced by exposure to 4% isoflurane and maintained at 1.5%. Subsequently, a local subcutaneous injection of xylocaine (7 mg/kg) in the head was performed. The mice were positioned in a stereotactic frame inside a Faraday cage for the whole duration of the procedure. For the acute implantation, a BSA-control microelectrode and a lactate-sensitive biosensor were stereotactically placed in the cortex of each hemisphere, following coordinates from a mouse brain atlas. An Ag/AgCl reference electrode was positioned under the skin of the neck.

Following the implantation of the enzymatic sensor under 1.5% isoflurane exposure, a bolus of medetomidine was subcutaneously injected. Isoflurane was gradually reduced to 1% over 10 minutes. Subsequently, medetomidine was continuously infused at a rate of 0.6 mg/kg/h, and isoflurane was gradually discontinued over the next 10 minutes. We decided to use medetomidine infusion during the recovery/stabilization period to mitigate potential risks associated with prolonged exposure to isoflurane, such as brain motion and bleeding at the site of implantation that may be caused by the vasodilatory properties of isoflurane. In this regard, medetomidine's

vasoconstrictive properties and sustained sedative effects helped ensure experimental stability during the recovery period, reducing the exposure to isoflurane before starting the recording. After 1 hour of signal stabilization, infusion was stopped and exposure to isoflurane was restored to 1%. Amperometric recording was performed during the following 30 minutes. Signal was collected using a VA-10M – Voltammetry/Amperometry module (npi, Germany) connected to a data acquisition system PowerLab/8SP (ADInstruments/ UK).

While the recording under 1% isoflurane starts more than 1.5h after buprenorphine bolus injection, making any residual effect on lactate metabolism presumably small, it starts shortly after stopping medetomidine infusion. Because medetomidine was not used before dMRS measurements, it is legitimate to wonder whether this might bias the comparison with dMRS. We therefore performed enzyme-electrode control measurements on three mice, by continuously recording extracellular lactate levels during an anesthesia protocol similar to the one used following electrode implantation for the APP/PS1 *versus* control experiments. It appears that extracellular lactate in the post-medetomidine 1%-isoflurane period returns to similar levels as in the pre-medetomidine 1%-isoflurane period, ruling out any persisting effect of medetomidine.

SI References

1. E. Mougel, S. Malaquin, M. Vincent, J. Valette, Using spectrally-selective radiofrequency pulses to enhance lactate signal for diffusion-weighted MRS measurements in vivo. *J Magn Reson* **334**, 107113 (2022).
2. I. Tkac, Z. Starcuk, I. Y. Choi, R. Gruetter, In vivo 1H NMR spectroscopy of rat brain at 1 ms echo time. *Magn Reson Med* **41**, 649-656 (1999).
3. W. T. Dixon, M. Sardashti, M. Castillo, G. P. Stomp, Multiple inversion recovery reduces static tissue signal in angiograms. *Magn Reson Med* **18**, 257-268 (1991).
4. C. Cudalbu *et al.*, Contribution of macromolecules to brain 1H MR spectra: Experts' consensus recommendations. *NMR Biomed* **34**, e4393 (2021).
5. S. W. Provencher, Estimation of metabolite concentrations from localized in vivo proton NMR spectra. *Magn Reson Med* **30**, 672-679 (1993).
6. C. Ligneul *et al.*, Diffusion-weighted magnetic resonance spectroscopy enables cell-specific monitoring of astrocyte reactivity in vivo. *Neuroimage* **191**, 457-469 (2019).
7. M. Palombo *et al.*, New paradigm to assess brain cell morphology by diffusion-weighted MR spectroscopy in vivo. *Proc Natl Acad Sci U S A* **113**, 6671-6676 (2016).
8. F. Sun, C. Dai, J. Xie, X. Hu, Biochemical issues in estimation of cytosolic free NAD/NADH ratio. *PLoS One* **7**, e34525 (2012).
9. V. Breukels *et al.*, Direct dynamic measurement of intracellular and extracellular lactate in small-volume cell suspensions with 13C hyperpolarised NMR. *NMR Biomed* **28**, 1040-1048 (2015).
10. F. Reineri, V. Daniele, E. Cavallari, S. Aime, Assessing the transport rate of hyperpolarized pyruvate and lactate from the intra- to the extracellular space. *NMR Biomed* **29**, 1022-1027 (2016).
11. F. Ahamed *et al.*, Modeling hyperpolarized lactate signal dynamics in cells, patient-derived tissue slice cultures and murine models. *NMR Biomed* **34**, e4467 (2021).
12. E. Mougel, J. Valette, M. Palombo, Investigating exchange, structural disorder, and restriction in gray matter via water and metabolites diffusivity and kurtosis time-dependence. *Imaging Neuroscience* **2**, 1-14 (2024).
13. E. Mougel, S. Malaquin, M. Palombo, J. Valette, Probing lactate exchange in gray matter via time-dependent DW-MRS. in *ISMRM Annual Meeting* (Toronto), p 683 (2023).
14. M. Palombo, C. Ligneul, J. Valette, Modeling diffusion of intracellular metabolites in the mouse brain up to very high diffusion-weighting: Diffusion in long fibers (almost) accounts for non-monoexponential attenuation. *Magn Reson Med* **77**, 343-350 (2017).
